# Supplementary material for: Dissipative quantum error correction and application to quantum sensing with trapped ions
Source: Nat Commun. 2017 Nov 28;8:1822. doi: 10.1038/s41467-017-01895-5 (PMC5704006; doi:10.1038/s41467-017-01895-5)
Supplement: Supplementary file 1 — Supplementary Information [file 41467_2017_1895_MOESM1_ESM.pdf]

## Supplementary Note 1 – Correction of other types of errors

In the following we discuss the generalization of our scheme to other types of errors. We start by discussing correlated spin-flips. Then we generalize the scheme to individual phase-flips, and finally explain how to correct for collective dephasing.

### Correlated spin-flips

The correction of the error syndromes $\sigma_{\mathrm{x}}^{(1)}$, $\sigma_{\mathrm{x}}^{(2)}$, and $\sigma_{\mathrm{x}}^{(3)}$ we have discussed in the main text does not interfere with each other and is performed using separate cooling resources. Instead of targeting individual errors, our scheme can as well be formulated to correct for correlated errors, such as, e.g., collective spin-flips,

$$L_{\mathrm{x}} = L_{\mathrm{x}}^{(1)} + L_{\mathrm{x}}^{(2)} + L_{\mathrm{x}}^{(3)} = \sqrt{\Gamma_{\mathrm{X}}}(\sigma_{\mathrm{x}}^{(1)} + \sigma_{\mathrm{x}}^{(2)} + \sigma_{\mathrm{x}}^{(3)}). \tag{1}$$

The action of such an error leaves the system in the state

$$|\psi_{\mathrm{X}}\rangle = \frac{1}{\sqrt{3}}(|\psi^{(1)}\rangle + |\psi^{(2)}\rangle + |\psi^{(3)}\rangle). \tag{2}$$

$|\psi\rangle$ can be restored by the single-qubit correcting operators in equations (6)–(8). In this case, however, a simpler strategy is to engineer a single correcting operator

$$L_{\mathrm{x,qec}} = L_{\mathrm{x,qec}}^{(1)} + L_{\mathrm{x,qec}}^{(2)} + L_{\mathrm{x,qec}}^{(3)}, \tag{3}$$

which is given by the sum of the operators in equations (6)–(8). We implement this interaction by replacing the individual mapping from single ions onto single auxiliary modes in equation (13) by a collective mapping of all ions onto a single auxiliary mode c, using the coupling

$$H_{\mathrm{aux},g} = g e^{i\delta_{\mathrm{c}} t} c^{\dagger} \sum_{j=1}^{3} (|0\rangle_j\langle e| + |1\rangle_j\langle f|) + H.c. \tag{4}$$

In the presence of only correlated noise, this approach is preferable since it reduces the required resources compared to realizing three operators. If both types, uncorrelated and correlated errors are present, the operations in equations (6)–(8) for the correction of uncorrelated errors suffice for the correction of both.

### Phase errors and collective dephasing

Instead of correcting for spin errors, our scheme can as well be formulated to correct for phase errors

$$L_{\mathrm{z}}^{(j)} = \sqrt{\Gamma_{\mathrm{z}}}\sigma_{\mathrm{z}}^{(j)}, \tag{5}$$

where $\sigma_{\mathrm{z}}^{(j)} = |0\rangle_j\langle 0| - |1\rangle_j\langle 1|$ applies a phase change to qubit $j$. To correct such errors we make the replacements $|0\rangle \to |+\rangle = (|0\rangle + |1\rangle)/\sqrt{2}$, $|1\rangle \to |-\rangle = (|0\rangle - |1\rangle)/\sqrt{2}$ throughout the protocol. Our codeword is then

$$|\psi_{\mathrm{z}}\rangle = c_{+}|+++\rangle + c_{-}|---\rangle, \tag{6}$$

and the states after an individual phase-flip are given by

$$|\psi_{\mathrm{z}}^{(j)}\rangle = \sigma_{\mathrm{z}}^{(j)}|\psi_{\mathrm{z}}\rangle, \tag{7}$$

e.g., $|\psi_{\mathrm{z}}^{(2)}\rangle = c_{+}|+-+\rangle + c_{-}|-+-\rangle$ for a phase-flip on the second of three qubits. Decay from $|+++\rangle$ and from $|---\rangle$ is again well-distinguishable. The codeword $|\psi_{\mathrm{z}}\rangle$ is recovered by the jump operators

$$L_{\mathrm{z,qec}}^{(1)} = \sqrt{\Gamma_{\mathrm{qec}}}\sigma_{\mathrm{z}}^{(1)} \frac{\mathbb{1} - \sigma_{\mathrm{x}}^{(1)}\sigma_{\mathrm{x}}^{(2)}}{2} \frac{\mathbb{1} - \sigma_{\mathrm{x}}^{(1)}\sigma_{\mathrm{x}}^{(3)}}{2} \tag{8}$$

$$L_{\mathrm{z,qec}}^{(2)} = \sqrt{\Gamma_{\mathrm{qec}}}\sigma_{\mathrm{z}}^{(2)} \frac{\mathbb{1} - \sigma_{\mathrm{x}}^{(1)}\sigma_{\mathrm{x}}^{(2)}}{2} \frac{\mathbb{1} - \sigma_{\mathrm{x}}^{(2)}\sigma_{\mathrm{x}}^{(3)}}{2} \tag{9}$$

$$L_{\mathrm{z,qec}}^{(3)} = \sqrt{\Gamma_{\mathrm{qec}}}\sigma_{\mathrm{z}}^{(3)} \frac{\mathbb{1} - \sigma_{\mathrm{x}}^{(1)}\sigma_{\mathrm{x}}^{(3)}}{2} \frac{\mathbb{1} - \sigma_{\mathrm{x}}^{(2)}\sigma_{\mathrm{x}}^{(3)}}{2} \tag{10}$$

with a correction rate $\Gamma_{\rm qec}$ for every single error. The operators are identical to the ones in equations (6)–(8) when making the above replacements. Experimentally, these modified operators can be realized by making similar replacements in all involved Hamiltonians. This can be achieved by a suitable combination of coupling lasers.

Another experimentally common error is correlated phase noise, or, collective dephasing. It is described by a single jump operator

$$L_{\rm Z} = L_{\rm z}^{(1)} + L_{\rm z}^{(2)} + L_{\rm z}^{(3)} = \sqrt{\Gamma_{\rm Z}}(\sigma_{\rm z}^{(1)} + \sigma_{\rm z}^{(2)} + \sigma_{\rm z}^{(3)}), \tag{11}$$

which causes a decay from $|\psi_{\rm z}\rangle$ to the single-error state

$$|\psi_{\rm Z}\rangle = \frac{1}{\sqrt{3}}(|\psi_{\rm z}^{(1)}\rangle + |\psi_{\rm z}^{(2)}\rangle + |\psi_{\rm z}^{(3)}\rangle). \tag{12}$$

To correct for this error, we use a coupling to a single auxiliary mode,

$$H_{{\rm aux},g} = g e^{i\delta_{\rm c} t} c^\dagger \sum_{j=1}^{3} (|+\rangle_j\langle e| + |-\rangle_j\langle f|) + H.c. \tag{13}$$

This realizes the jump operator

$$L_{Z,{\rm qec}} = L_{\rm z,qec}^{(1)} + L_{\rm z,qec}^{(2)} + L_{\rm z,qec}^{(3)}, \tag{14}$$

with the single-qubit correcting operators in Supplementary Equations (8)–(10).

## Supplementary Note 2 – Analysis

In this Supplementary Note we provide a detailed analysis of the presented scheme. We derive analytical expressions for the effective rates in the system and provide a numerical comparison to the full dynamics. Considering a simplified rate equation model we assess the performance of the scheme and determine the optimal parameters of the scheme analytically.

### Effective open system dynamics

Following the procedure of the effective operator formalism [1], we set up the non-Hermitian Hamiltonian $H_{\rm NH} = H_{\rm e} - iL_{\rm eng}^\dagger L_{\rm eng}/2$ describing the dynamics in the single-excitation manifold (higher excitations are neglected). It is of block-diagonal form

$$H_{\rm NH} = \sum_{\phi,f} H_{{\rm NH},|\phi\rangle|1\rangle_{\rm f}}. \tag{15}$$

Each of the blocks $H_{{\rm NH},|\phi\rangle|1\rangle_{\rm f}}$ contains a single motionally excited state $|\phi\rangle|1\rangle_{\rm f}$, where $|\phi\rangle$ can be any ground state of the system ions and $f \in \{{\rm a},{\rm b}\}$ denotes the motional mode. For example,

$$H_{{\rm NH},|100\rangle|1\rangle_{\rm a}} = \tilde{\Delta}|{\rm e}00\rangle|0\rangle_{\rm a}\langle 0|\langle {\rm e}00| + \tilde{\delta}|100\rangle|1\rangle_{\rm a}\langle 1|\langle 100| + G(|100\rangle|1\rangle_{\rm a}\langle 0|\langle {\rm e}00| + H.c.) \tag{16}$$

is needed for the correction of a single error on qubit 1,

$$H_{{\rm NH},|100\rangle|1\rangle_{\rm b}} = \tilde{\Delta}|\chi_{\rm f}\rangle|0\rangle_{\rm b}\langle 0|\langle \chi_{\rm f}| + \tilde{\delta}|100\rangle|1\rangle_{\rm b}\langle 1|\langle 100| + \sqrt{2}G|100\rangle|1\rangle_{\rm b}\langle 0|\langle \chi_{\rm f}| + H.c.) \tag{17}$$

participates in the undesired decay of $|100\rangle$, and

$$H_{{\rm NH},|111\rangle|1\rangle_{\rm a}} = \tilde{\Delta}|\xi_{\rm e}\rangle|0\rangle_{\rm a}\langle 0|\langle \xi_{\rm e}| + \tilde{\delta}|111\rangle|1\rangle_{\rm a}\langle 1|\langle 111| + \sqrt{3}G|111\rangle|1\rangle_{\rm a}\langle 0|\langle \xi_{\rm e}| + H.c.) \tag{18}$$

mediates the undesired decay from $|111\rangle$. The non-Hermitian Hamiltonian is formulated in terms of complex detunings $\tilde{\Delta} = \Delta - i\kappa_{\rm eng}/2 - i\gamma_m/2$ and $\tilde{\delta} = \delta - i\kappa_r/2$, where $\gamma_m$ ($\kappa_r$) accounts for potential finite lifetime of the excited levels (motional modes used for interrogation). Inversion of the blocks of the non-Hermitian Hamiltonian yields, e.g.,

$$\left(H_{{\rm NH},|100\rangle_{\rm a}}\right)^{-1} = \tilde{\Delta}_{1,{\rm eff}}^{-1}|{\rm e}00\rangle|0\rangle_{\rm a}\langle 0|\langle {\rm e}00| + \tilde{\delta}_{1,{\rm eff}}^{-1}|100\rangle|1\rangle_{\rm a}\langle 1|\langle 100| + \tilde{G}_{1,{\rm eff}}^{-1}\left(|100\rangle|1\rangle_{\rm a}\langle 0|\langle {\rm e}00| + H.c.\right) \tag{19}$$

$$\left(H_{{\rm NH},|100\rangle|1\rangle_{\rm b}}\right)^{-1} = \tilde{\Delta}_{2,{\rm eff}}|\chi_{\rm f}\rangle|0\rangle_{\rm a}\langle 0|\langle \chi_{\rm f}| + \tilde{\delta}_{2,{\rm eff}}|100\rangle|1\rangle_{\rm b}\langle 1|\langle 100| + \tilde{G}_{2,{\rm eff}}|100\rangle|1\rangle_{\rm b}\langle 0|\langle \chi_{\rm f}| + H.c.) \tag{20}$$

$$\left(H_{{\rm NH},|111\rangle_{\rm a}}\right)^{-1} = \tilde{\Delta}_{3,{\rm eff}}^{-1}|\xi_{\rm e}\rangle|0\rangle_{\rm a}\langle 0|\langle \xi_{\rm e}| + \tilde{\delta}_{3,{\rm eff}}^{-1}|111\rangle|1\rangle_{\rm a}\langle 1|\langle 111| + \tilde{G}_{3,{\rm eff}}^{-1}\left(|111\rangle|1\rangle_{\rm a}\langle 0|\langle \xi_{\rm e}| + H.c.\right) \tag{21}$$

with effective detunings and couplings

$$\tilde{\Delta}_{n,\mathrm{eff}} = \tilde{\Delta} - \frac{nG^2}{\tilde{\delta}}, \tag{22}$$

$$\tilde{\delta}_{n,\mathrm{eff}} = \tilde{\delta} - \frac{nG^2}{\tilde{\Delta}}, \tag{23}$$

$$\tilde{G}_{n,\mathrm{eff}} = \sqrt{n}G - \frac{\tilde{\Delta}\tilde{\delta}}{\sqrt{n}G}. \tag{24}$$

By our choice of $\Delta = \delta = G$ and the assumption $\kappa_{\mathrm{eng}} \gg \gamma_m, \kappa_r$, we engineer the effective detuning involved in the correction mechanism to be small and only limited by the linewidth due to the engineered cooling,

$$\tilde{\Delta}_{1,\mathrm{eff}} \approx -\frac{i\kappa_{\mathrm{eng}}}{2}. \tag{25}$$

In contrast, the terms with $n \neq 1$, i.e., $\tilde{\Delta}_{n,\mathrm{eff}} \approx (n-1)G$, are chosen to be large to render the undesired processes weak. We identify the driving fields with the perturbative couplings between ground and excited subspaces, $H_{\mathrm{drive},\Omega} = V_+ + V_-$, where $V_+$ is the part responsible for the excitation out of the ground subspace. We obtain the effective jump operators [1]

$$L_{\mathrm{eff}}^{(j)} = L_{\mathrm{eng}}^{(j)} H_{\mathrm{NH}}^{-1} V_+ = \sum_{n=1}^{3} \sqrt{\kappa_{\mathrm{eff},n}} \left( \sigma_-^{(j)} P_{n_1=n} + \sigma_+^{(j)} P_{n_0=n} \right). \tag{26}$$

Here, $P_{n_0=n}$ ($P_{n_1=n}$) denote projectors onto all ground states with $n$ atoms in state $|0\rangle$ ($|1\rangle$). The decay rates of the effective processes are given by

$$\kappa_{\mathrm{eff},n} = \frac{\kappa_{\mathrm{eng}}\Omega^2}{4|\tilde{\Delta}_{n,\mathrm{eff}}|^2}. \tag{27}$$

For the parameters at hand we obtain

$$\kappa_{\mathrm{eff},1} \approx \frac{\Omega^2}{\kappa_{\mathrm{eng}}}, \tag{28}$$

$$\kappa_{\mathrm{eff},2} \approx \frac{\kappa_{\mathrm{eng}}\Omega^2}{4G^2}, \tag{29}$$

$$\kappa_{\mathrm{eff},3} \approx \frac{\kappa_{\mathrm{eng}}\Omega^2}{16G^2}. \tag{30}$$

Here, $\kappa_{\mathrm{eff},1}$ is the effective decay rate from the single-error states to the logical subspace containing the codeword and $\kappa_{\mathrm{eff},2/3}$ are intrinsic loss rates: $\kappa_{\mathrm{eff},2}$ is the leakage rate from the single-error states to the double-error states and $\kappa_{\mathrm{eff},3}$ is a decay rate from the logical states to the single-error states. Comparing Supplementary Equation (26) with equation (9), thereby identifying $\Gamma_{\mathrm{qec}} = \kappa_{\mathrm{eff},1}$, we conclude that we have engineered the desired correction operators up to small terms $\mathcal{O}(\kappa_{\mathrm{eng}}\Omega^2/G^2)$ acting on manifolds other than the single-error subspace. In addition to the effective decays considered so far, the effective operator formalism [1] also contains an effective Hamiltonian $H_{\mathrm{eff}}$, which in the case at hand is found to only contain minor Stark shifts.

*Strong driving effects*

It should be noted that the above expressions are obtained by means of a perturbative formalism, and are thus only correct for $\Omega \ll \kappa_{\mathrm{eng}}$. Numerical optimization shows, however, that the scheme is more effective for $\Omega \sim \kappa_{\mathrm{eng}}$ because this allows for faster correction of errors (cf. Ref. [2]). We therefore include two strong driving effects: Power broadening and population of the excited states. Power broadening is relevant for the linewidth-limited process leading to $\kappa_{\mathrm{eff},1}$. Including it is easily achieved by replacing the bare linewidth of the excited states that mediate the correction of the single-error states, $\kappa_{\mathrm{eng}}$, by a broadened one, $\kappa_{\mathrm{eng}} + 2\Omega^2/\kappa_{\mathrm{eng}}$, which can be justified using adiabatic elimination [2]. We find

$$\kappa_{\mathrm{eff},1} = \frac{\kappa_{\mathrm{eng}}\Omega^2}{\kappa_{\mathrm{eng}}^2 + r\Omega^2} \tag{31}$$

for the preparation rate. While considering a simple two-level model would yield $r = 2$ [2], the numerics in the case at hand turn out to be more accurate for $r \approx 2.5$. This can be understood from further excited levels coupled by the drive. The error rates

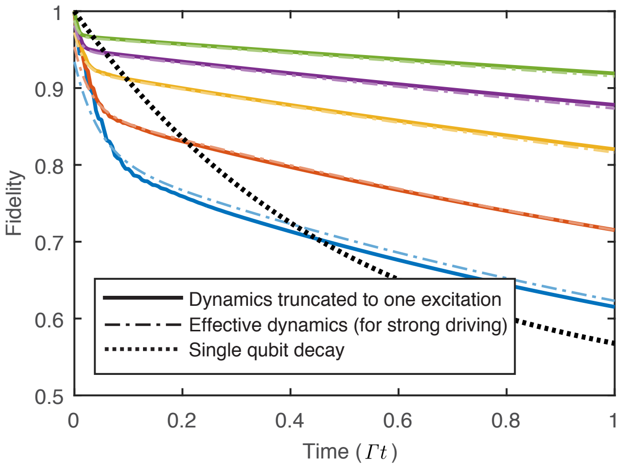


Supplementary Figure 1. Comparison of the effective and full dynamics for the presented protocol. We simulate the dynamics of the system subjected to spin-flip errors under the action of the error correction scheme for different ratios of the sideband coupling and the error rate, $G/\Gamma$ (from bottom to top: $G/\Gamma = 500, 1000, 2500, 5000, 10000$). We plot results obtained by simulating the full master equation restricted to at most one excitation (solid lines), and the effective dynamics of the ground states with strong driving corrections (dash-dotted lines). For each value of $G/\Gamma$, the fidelity at $t = 1/\Gamma$ is numerically optimized by the choice of $\kappa_{\rm eng}$ and $\Omega$. The results are compared with the corresponding decay of a single qubit (dotted line).

in Supplementary Equations (29)–(30) remain effectively unchanged by power broadening (under the reasonable assumption of $\Omega \ll G$). Secondly, we need to take into account that due to the increased driving, part of the population resides in the excited state manifold, which we previously eliminated. The main contribution to this steady population comes from the off-resonant excitation of the codeword. The effect can be taken into account by replacing the fidelity of the codeword $F$ by $(1-f_{\rm e})F$, where the excited fraction $f_{\rm e}$ is estimated to be [2]:

$$f_{\rm e} \approx \frac{(\sqrt{3}\Omega)^2}{(\sqrt{3}-1)^2 G^2} + \frac{(\sqrt{3}\Omega)^2}{(\sqrt{3}+1)^2 G^2} \approx \frac{6\Omega^2}{G^2}. \tag{32}$$

Here the expression $(\sqrt{3} \pm 1)^2 G^2$ has its origin in the energies $\pm\sqrt{3}G$ of the dressed states off-resonantly excited by the drives with detunings $G$.

*Numerical results and comparison of the methods*

We simulate the evolution due to the effective operators in Supplementary Equation (26), taking into account the strong driving effects in Supplementary Equations (31) and (32). The results after parameter optimization assuming sideband couplings $G/\Gamma = 500 - 10000$ are presented in Supplementary Figure 1. We plot them together with the result from a simulation of the full master equation in equation (23) truncated to a single excitation. We find that the effective dynamics agrees well with the full dynamics.

It can be seen that for $G/\Gamma = 1000$ and higher, substantial improvement is obtained compared to the unprotected single-qubit case.

**Rate equation model**

We reduce the complexity of the model further by reducing the effective dynamics to a system of rate equations. Here, we consider three subspaces: The logical subspace, with population $P_{\rm l}$, is comprised by the manifold of encoded states $|\psi\rangle = (c_0|000\rangle + c_1|111\rangle)/\sqrt{2}$. The correctable subspace, with population $P_{\rm c}$, contains the states where one error has occurred, $|\psi^{(j)}\rangle$. States where more than one qubit has been flipped form the subspace of uncorrectable states, $P_{\rm u}$. The described partitioning is possible due to the fact that the decay rates between the states of the considered subspaces are identical: all $|\psi^{(j)}\rangle$ decay to $|\psi\rangle$ with $\kappa_{\rm eff,1}$, and $|\psi\rangle$ decays to $|\psi^{(j)}\rangle$ with $\kappa_{\rm eff,3}$. In our analysis, we assume that we operate in the regime where $G \gg \Gamma$. This means that the dominant effect which limits the driving is the reduction of the pumping strength in Supplementary Equation (31), and we do not need to consider the population in the excited states. We also note that our simplified model does not capture

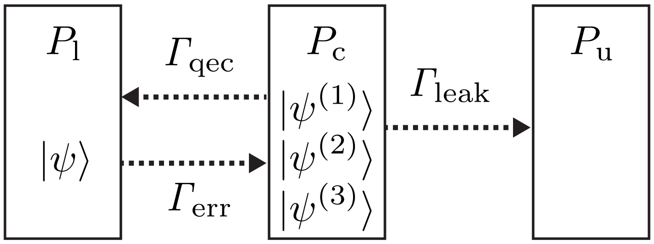


Supplementary Figure 2. Rate equation model. The dynamics can be described by three subspaces and the decay rates between them. The logical subspace (population $P_\mathrm{l}$) contains the codeword $|\psi\rangle$. It decays to the correctable subspace ($P_\mathrm{c}$) with the single-error states $|\psi^{(j)}\rangle$ ($j = 1, 2, 3$) at a rate $\Gamma_\mathrm{err}$. The error correction restores the logical subspace with a rate $\Gamma_\mathrm{qec}$. The single-error subspace decays to the subspace of uncorrectable states that contain more than one error (with population $P_\mathrm{u}$) at a rate $\Gamma_\mathrm{leak}$.

the steady state fidelities $\lim_{t\to\infty} F(t) > 0$. This is however of less concern here, where we are mainly interested in conditions for achieving a high fidelity. With the above assumptions we obtain a system of coupled differential equations,

$$\dot{P}_\mathrm{l} = -\Gamma_\mathrm{err} P_\mathrm{l} + \Gamma_\mathrm{qec} P_\mathrm{c}, \tag{33}$$

$$\dot{P}_\mathrm{c} = \Gamma_\mathrm{err} P_\mathrm{l} - (\Gamma_\mathrm{qec} + \Gamma_\mathrm{leak}) P_\mathrm{c}, \tag{34}$$

$$\dot{P}_\mathrm{u} = \Gamma_\mathrm{leak} P_\mathrm{c}, \tag{35}$$

with rates $\Gamma_\mathrm{err} = 3(\Gamma + \kappa_{\mathrm{eff},3})$, $\Gamma_\mathrm{qec} = \kappa_{\mathrm{eff},1}$ (neglecting that random spin-flips at a rate $\Gamma$ can also lead back to the desired state), and $\Gamma_\mathrm{leak} = 2(\Gamma + \kappa_{\mathrm{eff},2})$. The subspaces and transition rates are illustrated in Supplementary Figure 2. Solving for $P_\mathrm{l}(t)$ under the initial condition that $P_\mathrm{l}(0) = 1$ yields

$$F(t) = P_\mathrm{l}(t) = e^{-\Gamma_s t}\left[1 - E_0\left(1 - e^{-\Gamma_\mathrm{f} t}\right)\right]. \tag{36}$$

The evolution of the logical population is thus dominated by two exponential decays, a fast one with the rate

$$\Gamma_\mathrm{f} = \sqrt{\Gamma_\mathrm{total}^2 - 4\Gamma_\mathrm{err}\Gamma_\mathrm{leak}}, \tag{37}$$

where $\Gamma_\mathrm{total} = \Gamma_\mathrm{qec} + \Gamma_\mathrm{err} + \Gamma_\mathrm{leak}$ and a slow one with rate

$$\Gamma_s = \frac{1}{2}(\Gamma_\mathrm{total} - \Gamma_\mathrm{f}) \approx \frac{\Gamma_\mathrm{err}\Gamma_\mathrm{leak}}{\Gamma_\mathrm{qec}}. \tag{38}$$

For intermediate times $1/\Gamma_\mathrm{f} \ll t \sim 1/\Gamma$, the exponential decays appear linear, as seen in Supplementary Figure 1. The two timescales $\Gamma_\mathrm{f}$ and $\Gamma_s$ thus generate the characteristic "hockey stick" form of the curve that can be seen from Fig. 5. Assuming the drop due to $\Gamma_\mathrm{f}$ to be faster than the timescale of interest, we can simplify Supplementary Equation (36) to

$$F(t) = P_\mathrm{l}(t) = e^{-\Gamma_\mathrm{eff} t}(1 - E_0). \tag{39}$$

In this simplified model, the evolution is described by the two quantities:

$$E_0 = \frac{\Gamma_\mathrm{err} - \Gamma_\mathrm{eff}}{\Gamma_\mathrm{f}} \approx \frac{\Gamma_\mathrm{err}}{\Gamma_\mathrm{qec}}, \tag{40}$$

$$\Gamma_\mathrm{eff} = \Gamma_\mathrm{leak} E_0. \tag{41}$$

Here $E_0$ denotes the static error of the scheme that arises at approximately $t = 0$, when the scheme is switched on. The physical explanation for this initial drop is that $P_\mathrm{c}$ and $P_\mathrm{l}$ come to equilibrium fast and then population slowly leaks from this coupled subspace. Alternatively it can be understood from the fact that it takes a certain time to pump back after a spin-flip. The effective decay rate $\Gamma_\mathrm{eff}$, previously $\Gamma_\mathrm{s}$, is the slow effective decay rate of the codeword when it is protected by the error correction scheme.

### Minimization of the error and the decay rate

Using the solution of the rate equation model, we can optimize the fidelity $F = P_\mathrm{l}(t)$ in Supplementary Equation (39) at a given time $t$ by the choice of available parameters $\kappa_\mathrm{eng}$ and $\Omega$. For that we need to fulfill the condition:

$$\frac{\partial F}{\partial x} = -\left(\frac{\partial E_0}{\partial x} + (1 - E_0)\frac{\partial \Gamma_\mathrm{eff}}{\partial x} t\right) e^{-\Gamma_\mathrm{eff} t} = 0, \tag{42}$$

for $x \in \{\kappa_{\rm eng}, \varOmega^2\}$. For the derivatives we have

$$\frac{\kappa_{\rm eng}}{E_0}\frac{\partial E_0}{\partial \kappa_{\rm eng}} \approx \frac{\kappa_{\rm eng}\varOmega^2}{16\varGamma G^2 + \kappa_{\rm eng}\varOmega^2} + \frac{\kappa_{\rm eng}^2 - r\varOmega^2}{\kappa_{\rm eng}^2 + r\varOmega^2}, \tag{43}$$

$$\frac{\varOmega^2}{E_0}\frac{\partial E_0}{\partial \varOmega^2} \approx \frac{\kappa_{\rm eng}\varOmega^2}{16\varGamma G^2 + \kappa_{\rm eng}\varOmega^2} - \frac{\kappa_{\rm eng}^2}{\kappa_{\rm eng}^2 + r\varOmega^2}, \tag{44}$$

$$\frac{x}{\varGamma_{\rm eff}}\frac{\partial \varGamma_{\rm eff}}{\partial x} \approx \frac{x}{E_0}\frac{\partial E_0}{\partial x} + \frac{\kappa_{\rm eng}\varOmega^2}{4\varGamma G^2 + \kappa_{\rm eng}\varOmega^2}. \tag{45}$$

By setting these derivatives to zero and comparing Supplementary Equations (43) and (44), and (45) for $x = \kappa$ and $x = \varOmega^2$, we find that

$$\varOmega_{\rm opt} = \sqrt{\frac{2}{r}}\kappa_{\rm eng,opt} \approx 0.9\ \kappa_{\rm eng,opt}, \tag{46}$$

where, in the second step, we have used $r = 2.5$. To solve for $\kappa_{\rm eng,opt}$, we make an ansatz $\kappa_{\rm eng,opt}\varOmega_{\rm opt}^2 = \alpha\varGamma G^2$ With this, Supplementary Equation (42) can be written as

$$(2 + 5\tau_A)\alpha^2 + 8\tau_A(\tau_A - 1) - 64(\tau_A + 1) = 0, \tag{47}$$

where $\tau_A = A\tau_{\rm eff}$ with $A = (1 - E_0)/E_0$ and $\tau_{\rm eff} = \varGamma_{\rm eff}t$. From this we derive the condition

$$\alpha = \frac{-4[(5\tau_A - 1) + 3\sqrt{5\tau_A^2 + 2\tau_A + 1}]}{2 + 5\tau_A}. \tag{48}$$

Note that we neglected one solution because of the requirement $\alpha > 0$. The optimal decay rate is then given by

$$\kappa_{\rm eng,opt} \approx \sqrt[3]{\frac{r\ \alpha}{2}\ \varGamma G^2}. \tag{49}$$

Finally, this leads to the optimal static error and effective decay rate of the form

$$E_0 \approx \beta_{\rm E}\left(\frac{\varGamma}{G}\right)^{2/3}, \tag{50}$$

$$\varGamma_{\rm eff} \approx \beta_{\varGamma}\left(\frac{\varGamma}{G}\right)^{2/3}\varGamma, \tag{51}$$

with the numeric prefactors $\beta_{\rm E}$ and $\beta_{\varGamma}$ being given by

$$\beta_{\rm E}(\tau_A) = \frac{9}{16}(16 + \alpha)\left(\frac{\beta^2}{4\alpha}\right)^{1/3}, \tag{52}$$

$$\beta_{\varGamma}(\tau_A) = \frac{9}{32}(16 + \alpha)(4 + \alpha)\left(\frac{\beta^2}{4\alpha}\right)^{1/3}. \tag{53}$$

We thus find that the optimal parameters depend on the operation time of the protocol. Here, we regard two limiting cases: First, we consider the long-time limit where $\tau_A \gg 1$. This corresponds to optimizing $\varGamma_{\rm eff}$ under the assumption that $\varGamma_{\rm eff}t \gg E_0$. This yields $\alpha_\infty \approx 4(3\sqrt{5} - 5)/5 \approx 1.4$ and thereby results in an optimal decay rate $\kappa_{\rm eng,opt} \approx 1.2\sqrt{\varGamma G^2}$. For the correction rate, we obtain $\varGamma_{\rm qec} = 2\kappa/(3\beta) \approx 0.3\sqrt[3]{\varGamma G^2}$, for the error and leakage rates $\varGamma_{\rm err} \approx 3.3\ \varGamma$ and $\varGamma_{\rm leak} \approx\ 2.7\ \varGamma$. For the prefactors in Supplementary Equations (50)–(51) we thus find $\beta_{\rm E} \approx 10$ and $\beta_{\varGamma} \approx 22$.

Alternatively, the protocol can be optimized for short operation times, $\tau_A \to 0$. Here, minimizing the initial drop $E_0$ requires a stronger correction rate, and thus, higher $\kappa_{\rm eng}$, at the expense of a higher leakage rate. We obtain $\alpha_0 = 8$, resulting in $\kappa_{\rm eng} \approx 2.2\sqrt[3]{\varGamma G^2}$, $\varGamma_{\rm qec} \approx 0.6\sqrt[3]{\varGamma G^2}$, $\varGamma_{\rm err} = 9/2\ \varGamma$, and $\varGamma_{\rm leak} = 6\ \varGamma$. This yields the coefficients $\beta_{\rm E} \approx 8$ and $\beta_{\varGamma} = 47$. The reduction of the initial drop by this parameter choice is therefore not very pronounced ($\beta_{\rm E} = 8$ vs. $\beta = 10$), whereas the slope of the effective decay is strongly increased ($\beta_{\varGamma} = 22$ vs. $\beta_{\varGamma} = 47$). The optimization for $\tau_A \gg 1$ hence constitutes the better choice and is used for the metrology application.

The above findings for $\kappa_{\rm eng,opt}$ in the long-time limit are in good agreement with the numerical optimization of this parameter from the truncated dynamics which fulfills $\tau_A \gg 1$. Using $\beta = 2.5$, the optimal driving strength in Supplementary Equation (46) is, however, slightly stronger than the result obtained from the numerical optimization, where we find $\varOmega_{\rm opt} \approx 4\kappa_{\rm eng,opt}/5$. This is due to the fact that we ignored the population in the excited states: This amounts to about $4 - 7\%$ at $t = 1/\varGamma$ for typical parameters and would thus yield a smaller value for $\varOmega_{\rm opt}$. The resulting discrepancy vanishes, however, in the limit of large $G/\varGamma$.

## Supplementary Note 3 – Application to quantum metrology: Extension to $N_{\log}$ logical qubits

Here we consider an extension of the metrology scheme discussed in the main text to $N_{\log}$ logical qubits, using a segmented trap [3, 4]. In this setup, groups of three ions constitute logical qubits that are trapped in separate trap segments. For sufficiently high potential barriers separating the ion triples, the motional modes associated with the different logical qubits can be assumed to be independent. The Ramsey sequence explained in the main text can be straightforwardly generalized to this setting, as explained in the following.

Ramsey scheme using a product state of $N_{\log}$ logical qubits:

(i) Starting from the initial state $|0\rangle_{\rm L}^{\otimes N_{\log}} = |000\rangle^{\otimes N_{\log}}$, individual Ramsey pulses are applied to the logical qubits to prepare the state $|\psi(0)\rangle = |+\rangle_{\rm L}^{\otimes N_{\log}}$ with $|+\rangle_{\rm L} = (|000\rangle + |111\rangle)/\sqrt{2}$.

(ii) During the Ramsey waiting time of duration $\tau_{\rm R}$, each logical qubit state picks up a relative phase $\phi(\tau_{\rm R}) = 3\omega\tau_{\rm R}$, such that $|\psi(\tau_{\rm R})\rangle = \big((|000\rangle + e^{-i3\omega\tau_{\rm R}}|111\rangle)/\sqrt{2}\big)^{\otimes N_{\log}}$.

(iii) A second set of individual Ramsey pulses that acts on each logical qubit separately, transforms the evolved state into $|\psi'(\tau_{\rm R})\rangle = (\cos(3\omega\tau_{\rm R}/2)|111\rangle + i\sin(3\omega\tau_{\rm R}/2)|000\rangle)^{\otimes N_{\log}}$.

(iv) For each logical qubit (i.e. for each trap segment), a measurement on one of the three physical qubits is performed in the $\sigma_{\rm z}$ basis. For each ion triple, the probability to detect the first (or any other) qubit in state $|1\rangle$ is given by $P_1 = \cos^2(3\omega\tau_{\rm R}/2)$, which allows one to determine the phase $\phi(\tau_{\rm R})$ and therefore the signal strength $\omega$.

In the ideal case, the sensitivity of this measurement[1] is given by $|\delta\omega| = 1/(3\sqrt{N_{\log}\tau_{\rm R}T})$, where $T = n_{\rm runs}\tau_{\rm R}$ is the total measurement time. As described in the main text, the dependence of the measurement precision $|\delta\omega|$ on the Ramsey time $\tau_{\rm R}$ changes in the presence of noise. The limitations due to transversal noise can be mitigated by applying our quantum error correction scheme. Since the protocol is applied for each trap segment individually, the results discussed in the main text generalize directly to the setting involving $N_{\log}$ logical qubits.

We note that the three-qubit Ramsey pulses in step (iii) of the scheme can be replaced by individual pulses that act on each physical qubit separately, $|0\rangle \to (|0\rangle + |1\rangle)/\sqrt{2}$, $|1\rangle \to (|1\rangle - |0\rangle)/\sqrt{2}$. The phase $\phi(\tau_{\rm R})$ and therefore the signal strength $\omega$ can then be inferred by measuring the parity operator $P_z = \sigma_{\rm z}^{(1)} \otimes \sigma_{\rm z}^{(2)} \otimes \sigma_{\rm z}^{(3)}$ for each logical qubit (which can be implemented straightforwardly by measuring the state of each individual qubit in the $\sigma_{\rm z}$-basis). In the ideal case, the sensitivity of this modified scheme is also given by $|\delta\omega| = 1/(3\sqrt{N_{\log}\tau_{\rm R}T})$.

The Ramsey sequence described above does not yield a quantum enhancement of the scaling of the measurement precision $|\delta\omega|$ with the number of logical qubits $N_{\log}$ since the logical qubits remain in a product state throughout the measurement sequence. To conclude this section, we consider an alternative setting where collective effects with respect to the logical qubits play a role and where quantum error correction allows one in principle to perform Heisenberg limited precision measurements even in the presence of noise. This setting is conceptually interesting even though its applicability may be limited in practice. More specifically, we consider the following Ramsey sequence assuming a trap with adjustable segmentation.

Ramsey scheme using $N_{\log}$ entangled logical qubits:

(i) The ions are initially held in an unsegmented trap and couple to a common motional mode which allows one to apply a global Ramsey pulse transforming the initial state $|0\rangle_{\rm L}^{\otimes 3N_{\log}}$ into $|\psi(0)\rangle = (|0\rangle^{\otimes 3N_{\log}} + |1\rangle^{\otimes 3N_{\log}})/\sqrt{2}$.

(ii) After the first Ramsey pulse, potential barriers are ramped up which divide the trap into $N_{\log}$ independent trap segments that each contain a logical qubit. This segmentation into sets of three ions is kept during the whole Ramsey waiting period $\tau_{\rm R}$ and the quantum error correction scheme is applied for each segment individually. Ideally, the relative phase acquired during the Ramsey waiting time results in $|\psi(\tau_{\rm R})\rangle = (|0\rangle^{\otimes 3N} + e^{-i3N_{\log}\omega\tau_{\rm R}}|1\rangle^{\otimes 3N})/\sqrt{2}$.

(iii) After the Ramsey waiting period, the potential barriers of the trap are ramped down such that the ions share again a motional mode that can be used to perform a second global Ramsey pulse resulting in the state $|\psi'(\tau_{\rm R})\rangle = \cos(3N_{\log}\omega\tau_{\rm R}/2)|1\rangle^{\otimes 3N_{\log}} + i\sin(3N_{\log}\omega\tau_{\rm R}/2)|0\rangle^{\otimes 3N_{\log}}$.

---

[1] In the case considered here, the number of experimental data is given by $n_{\rm data} = N_{\log}\cdot n_{\rm runs} = N_{\log}T/\tau_R$. Evaluating $\Delta P_1$ and $dP_1/d\omega$ as described in the Methods Section yields therefore $|\delta\omega| = 1/(3\sqrt{N_{\log}\tau_{\rm R}T})$.

(iv) As last step, one of the $3N_{\log}$ qubits is measured in the $\sigma_z$ basis.

In the absence of imperfections, the sensitivity is given by $\sqrt{T}|\delta\omega| = 1/(N\sqrt{\tau_R})$, where the $N = 3N_{\log}$ is the number of ions. If we consider a situation involving only the signal Hamiltonian and spin-flips at a rate $\Gamma$ (in the absence of error correction), the measurement precision drops to $\sqrt{T}|\delta\omega| = c\Gamma^{1/6}/N^{5/6}$, where $c$ is a numerical constant that depends on the estimated parameter $\omega$ (see [5, 6]). If a continuous three-qubit repetition code is operated in the strong correction regime where the correction rate $\Gamma_{\rm qec}$ is much larger than the spin-flip rate $\Gamma$, the dynamics can be described in terms of logical qubits that undergo logical spin-flips $|000\rangle \leftrightarrow |111\rangle$ at a reduced rate $\Gamma_{\rm L}$. In this regime, the population of states involving one or two single-qubit spin-flips becomes negligible [7]. As a result, the achievable measurement precision is given by $\sqrt{T}|\delta\omega| \propto \Gamma_{\rm L}^{1/6}/N^{5/6}$, where $\Gamma_{\rm L} \propto \epsilon\Gamma$ with $\epsilon = \Gamma/\Gamma_{\rm qec}$. By scaling up the correction rate with the number of qubits such that $\epsilon \propto N^{-1/6}$, the measurement precision of the scheme is Heisenberg limited (up to a maximum number of logical qubits $N_{\log,\max}$ that is determined by the maximum achievable error correction rate). While it is conceptually interesting that the Heisenberg scaling can be restored in principle, experimental imperfections will considerably limit its applicability. Still, the Ramsey scheme based on a product state of $N_{\log}$ logical qubits described above is likely to be of higher practical value for current quantum hardware.
We note that the $N$-qubit Ramsey pulse in step (iii) of the Heisenberg limited measurement scheme described above can be replaced by pulses that act on each logical qubit separately $|0\rangle_{\rm L} \to (|0\rangle_{\rm L} + |1\rangle_{\rm L})/\sqrt{2}$, $|1\rangle_{\rm L} \to (|1\rangle_{\rm L} - |0\rangle_{\rm L})/\sqrt{2}$. In this case, a measurement of the logical parity operator $P_{z_{\rm L}} = \sigma_{\rm z,L}^{(1)} \otimes ... \otimes \sigma_{\rm z,L}^{(N_{\log})}$ (which can be realized by measuring the states of all physical qubits locally in the $\sigma_z$-basis) allows ideally also for a Heisenberg limited measurement of of the signal strength $\omega$ [6].

## Supplementary Note 4 – External Imperfections

### Imperfect cooling and heating of the motion

We investigate the effect of imperfections due to imperfect cooling and heating of the motional modes by simulating the master equation including states with up to two motional excitations per mode (including three did not change the results noticeably). We do not perform adiabatic elimination of the modes $\mathrm{c}^{(j)}$. Instead, we set up the couplings $H_{\mathrm{drive},\Omega}$ and $H_{\mathrm{aux},\Omega}$ in a time-dependent manner, with two frequency tones, a red-detuned ($+G$) one and a blue-detuned one ($-G$), which also improves the fidelity. The heating is modeled by operators $L_r = \sqrt{\kappa_r^+}r^\dagger$ where $r \in \{\mathrm{a}, \mathrm{b}, \mathrm{c}^{(j)}\}$ for $j = 1, 2, 3$. For each mode $r$, we assume an initial thermal distribution described by a mean phonon number $\bar{n}_r = \kappa_r^+/(\kappa_r + \kappa_r^+)$.

For the auxiliary modes $\mathrm{c}^{(j)}$ we find that both imperfect cooling and heating only lead to minor reductions of the fidelity at the sub-percent level. For example, for $\bar{n}_r \sim 0.01$ and $\kappa_r^+ \sim 10$ quanta$/s$ for each mode [8, 9], and $G/\Gamma = 1000$, we obtain a reduction of $0.003$ at $t = 1/\Gamma$. This can be understood from the fact that the cooling of the motional modes $L_\kappa$ is assumed to be strong also in the absence of imperfections, in order to remove errors from the system. The optimal cooling rate is found to be $\kappa \sim G$, which, for $G/(2\pi) \sim 10$ kHz translates into $\kappa/(2\pi) \sim 10$ kHz. Such rates can be realized using continuous sympathetic cooling. If ancilla ions are used rather than auxiliary modes, these need to be continuously reset at a rate $\kappa$.

For the interrogation modes a and b, states with motional excitations are not supposed to participate in the scheme. Hence, the resulting error due to imperfect cooling can be estimated by the population in higher motional states, $P_{n>0}$. Assuming $\bar{n} \ll 1$, this is approximately given by $P_{n>0} \approx \bar{n}_{\rm a} + \bar{n}_{\rm b} \approx 2\bar{n}$. Again, this error is small if the interrogation modes are cooled.

Motional heating of the interrogation modes has a more pronounced effect on the scheme than for the auxiliary modes. This is due to the fact that $H_{\mathrm{int},G}$ is assumed to be strong ($G \gg \Gamma$), in order to create the splittings of the resonances. Excitations of the modes a and b can thus couple to excitations of the system ions, leading to additional loss channels (cf. [10]) causing errors. Cooling of the interrogation modes can counteract these effects. However, the cooling rate $\kappa$ should be moderate, to minimize effects on the coherence of the two paths of the error correction which involve both modes, a and b. Considering $G/\Gamma = 1000$, assuming for each mode a heating rate $\kappa_r^+ = 3$ quanta$/s$ (as recently demonstrated in [9]), a cooling rate $\kappa_{\rm a/b} = G/1000$, and the corresponding thermal distribution with $\bar{n} \approx 0.05$, we observe a drop from $F = 0.81$ to $F = 0.74$ for $t = 1/\Gamma$.

As expected, the additional error due to heating of the interrogation modes is thus larger than that due to heating of the auxiliary modes, and is comparable to the initial drop of $E_0 \approx 0.1$. Yet, the operation of the error-correcting scheme still allows for much higher fidelity than in the uncorrected single-ion case with $F \approx 0.56$ at $t = 1/\Gamma$ and is thus useful for quantum metrology applications. The fidelity can be increased by using stronger sideband couplings compared to the error rate. To further suppress losses due to motional decoherence, the cooling of the interrogation modes and the operation of the error correction scheme can be separated in time.

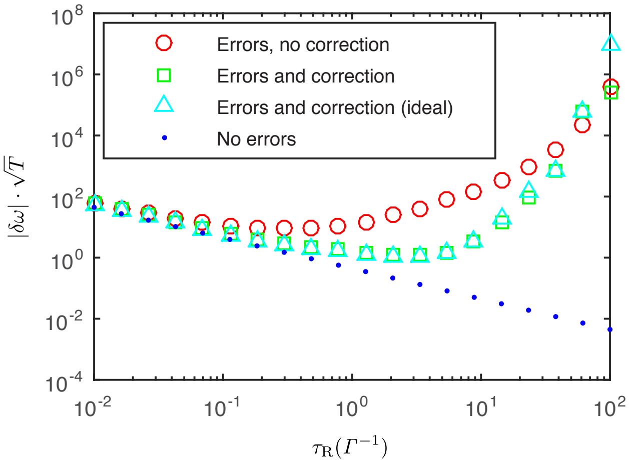


Supplementary Figure 3. Quantum error correction enhanced sensitivity in the presence of parallel noise. The plots show the normalized measurement precision $|\delta\omega|\sqrt{T}$ of a spin-flip corrected Ramsey measurement in the presence of additional phase noise. As a consequence of the uncorrected noise, the prolongation of the Ramsey time becomes detrimental for longer times $\Gamma\tau_{\mathrm{R}} \gtrsim 10^2$, but is still beneficial for $\Gamma\tau_{\mathrm{R}} \sim 10^0$. In the simulation, we use $G = 5000\,\Gamma$, $\Gamma_{\mathrm{Z}} = \Gamma/100$, where $\Gamma_{\mathrm{Z}}$ ($\Gamma$) is the collective phase-flip (individual spin-flip) rate, and the optimized parameters $\Omega = 4\kappa_{\mathrm{eng}}/5$ and $\kappa_{\mathrm{eng}} = 1.2\sqrt[3]{\Gamma G^2}$.

### Sensitivity in the presence of parallel noise

In our discussion of the sensitivity of the quantum measurement protected by our error-correcting scheme, we have so far considered the correction of local spin flips

$$L_{\mathrm{x}}^{(j)} = \sqrt{\Gamma}\sigma_{\mathrm{x}}^{(j)}, \quad j = 1, 2, 3, \tag{54}$$

with a rate $\Gamma$. In the following, we investigate the effect of collective phase noise as an additional, experimentally common source of noise, described by a jump operator

$$L_{\mathrm{Z}} = \sqrt{\Gamma_{\mathrm{Z}}}(\sigma_{\mathrm{z}}^{(1)} + \sigma_{\mathrm{z}}^{(2)} + \sigma_{\mathrm{z}}^{(3)}), \tag{55}$$

with a rate $\Gamma_Z$. Such noise acting along the direction of the signal cannot be targeted by error correction. Supplementary Figure 3 shows the improvement of the measurement precision that can be achieved in the presence of both transversal and parallel noise ($\sigma_{\mathrm{x}}$ and $\sigma_{\mathrm{z}}$ errors). It can be seen that the performance of the scheme is reduced compared to the case without parallel noise shown in Fig. 7. For long Ramsey times $\Gamma\tau_{\mathrm{R}} \gtrsim 10^2$, the application of the error correction results in a reduction of the sensitivity, which turns out to be stronger for the idealized operators than for the full dynamics in some cases. Still, for times $\Gamma\tau_{\mathrm{R}} \sim 10^0$, using the error correction scheme leads to significantly better results than in the cases where the scheme is not applied.

---

### References


[1] Reiter, F. & Sørensen, A. S. Effective operator formalism for open quantum systems. *Phys. Rev. A* **85,** 032111 (2012).

[2] Reiter, F., Reeb, D. & Sørensen, A. S. Scalable preparation of many-body entanglement. *Phys. Rev. Lett.* **117,** 040501 (2016).

[3] Wunderlich, H., Wunderlich, C., Singer, K. & Schmidt-Kaler, F. Two-dimensional cluster-state preparation with linear ion traps. *Phys. Rev. A* **79,** 052324 (2009).

[4] Alonso, J., Leupold, F. M., Keitch, B. C. & Home, J. P. Quantum control of the motional states of trapped ions through fast switching of trapping potentials. *New J. Phys.* **15,** 023001 (2013).

[5] Chaves, R., Brask, J. B., Markiewicz, M., Kołodyński, J. & Acin, A. Noisy Metrology beyond the Standard Quantum Limit. *Phys. Rev. Lett.* **111,** 120401 (2013).

[6] Brask, J.B., Chaves, R., & Kołodyński, J. Improved Quantum Magnetometry beyond the Standard Quantum Limit. *Phys. Rev. X* **5,** 031010 (2015).

[7] Ippoliti, M., Mazza, L., Rizzi, M. & Giovannetti, V. A perturbative approach to continuous-time quantum error correction. *Phys. Rev. A* **91,** 042322 (2015).

[8] Schindler, P. *et al.* A quantum information processor with trapped ions. *New J. Phys.* **15,** (2013).
[9] Hempel, C. *Digital quantum simulation, Schrödinger cat state spectroscopy and setting up a linear ion trap*, PhD thesis, Leopold-Franzens-Universität Innsbruck (2014).
[10] Lin, Y. *et al.* Dissipative production of a maximally entangled steady state of two quantum bits. *Nature* **504,** 415–418 (2013).
